# Active Shingles Infection as Detected on ^18^F-FDG PET/CT

**DOI:** 10.3389/fonc.2013.00103

**Published:** 2013-04-24

**Authors:** Razi Muzaffar, Mark Fesler, Medhat M. Osman

**Affiliations:** ^1^Division of Nuclear Medicine, Department of Radiology, Saint Louis UniversitySt. Louis, MO, USA; ^2^Department of Hematology/Oncology, Saint Louis UniversitySt. Louis, MO, USA; ^3^Division of Nuclear Medicine, Department of Radiology, St. Louis VA Medical CenterSt. Louis, MO, USA

**Keywords:** *Varicella zoster*, shingles, herpes zoster, dermatome, PET/CT

## Abstract

We present the case of a 56-year-old male with a history of recurrent follicular lymphoma undergoing chemotherapy with multiple ^18^F-FDG PET-CT studies at an outside facility. He developed a painful erythematous, pruritic rash in the left back requiring a visit to the emergency room. He was diagnosed and treated for *Varicella zoster* infection. He then presented to our imaging center 2 months later for a follow up ^18^F-FDG PET/CT study. Imaging demonstrated a cutaneous band of increased metabolic activity in the upper back following a dermatomal distribution. This was confirmed to be in the same area as the treated *Varicella zoster* eruption. A subsequent follow up ^18^F-FDG PET-CT scan 4 months later to confirm tumor resolution demonstrated the abnormal band of uptake in the back had resolved. This case illustrates the significance of being aware of this entity and to distinguish it from metastasis, especially in patients with a known history of malignancy.

## Case Presentation

**Figure 1:**
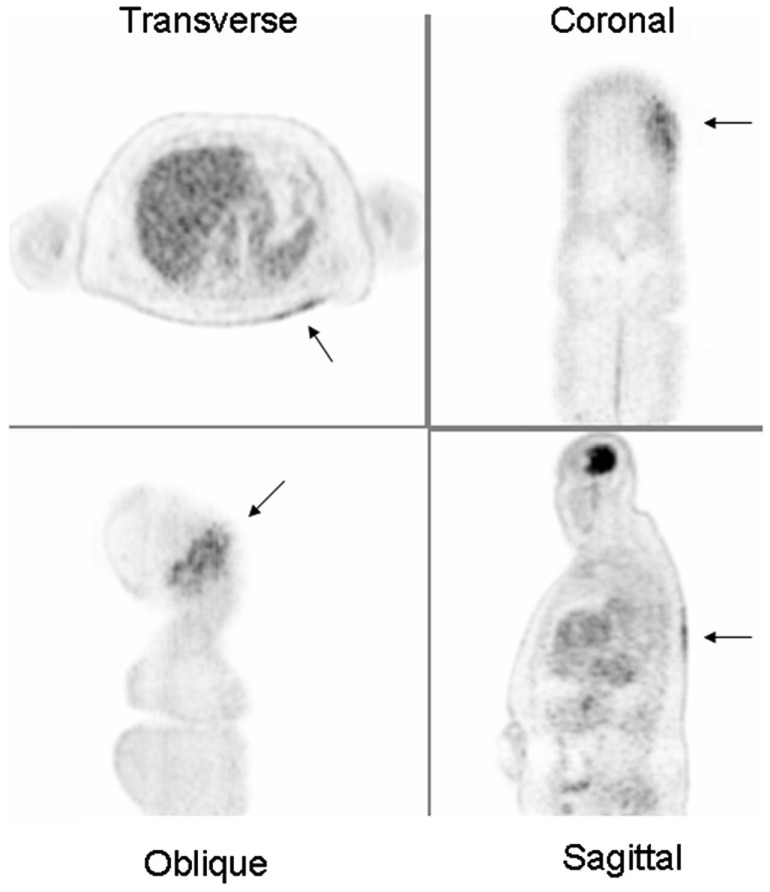
**^18^FDG PET images demonstrating a dermatomal distribution of increased metabolic activity in the left upper back (arrows)**. These findings were consistent with active *Varicella zoster* infection clinically.

A 56-year-old male with a history of stage IIIA follicular lymphoma initially diagnosed 2 years prior to arriving at our imaging facility as a follow up to treatment. He was initially treated with an R-CVP (Rituximab, Cyclophosphamide, Vincristine, and Prednisone) chemotherapy regimen and attributed a complete response. However, he had evidence of relapse a year later. He was then placed on a MOPP-R regimen (Mustargen, Oncovin, Procarbazine, Prednisone, and Rituximab) and had multiple ^18^F-FDG PET-CT studies at an outside facility. The most recent PET-CT from 6 months earlier showed no evidence of malignancy. Since the patient had a history of recurrence, a repeat PET-CT scan to document stability was performed at our facility which demonstrated a superficial band of increased metabolic activity in the left mid back following a dermatomal distribution and associated mild skin thickening (see Figure 1). The managing oncologist informed us that the patient had gone to the emergency room 2 months prior to the PET-CT. He presented there with a painful erythematous, pruritic rash in the left mid back following the T10-12 dermatome. The emergency room physician diagnosed the patient with *Varicella zoster* and treated him with pain medication and valacyclovir 1000 mg orally three times per day for 7 days.

Two weeks after the emergency room visit, he returned to his oncologist for a follow up appointment and physical examination found crusted lesions in the area of the rash. The patient continued to have neuropathic pain in the area and was taking oxycodone for relief. He had been prescribed valacyclovir 500 mg orally twice daily as prophylaxis prior to the outbreak, but he admit to not being adherent. The patient returned to our imaging facility 4 months after the previous PET-CT and the superficial band of increased metabolic activity in the left mid back had resolved. A month later the patient returned to his oncologist with no signs of a rash.

## Discussion

*Varicella zoster* is a common but serious infection in immunocompromised patients, especially in those with deficient cell-mediated immunity. However, it is often effectively prophylaxed with antivirals in these patients. The infection typically manifests as a painful rash often limited to a skin dermatome but can spread causing more serious complications and become life threatening. Active infection will show increased metabolic activity in the inflammatory cells on FDG PET images.

Active *Varicella zoster* infection is not routinely seen on PET-CT in our experience. There are only a few case reports describing this uncommon entity (Joyce and Carlos, 2006; Nair and Al Shemmari, 2011). However, none of these cases demonstrate a dermatomal distribution, as seen in our case, which may help in differentiating it from cutaneous metastasis. Valacyclovir is commonly used in the treatment of infection in immunocompromised patients and has been found to reduce the incidence of herpesviridae infection (Anderson et al., 1984; Lee et al., 2012).

## Conclusion

Active infection with *Varicella zoster* virus may display increased metabolic activity in the inflammatory cells on PET. This case illustrates the significance of being aware of this entity and to distinguish it from metastasis, especially in patients with known malignancy (Castellucci et al., 2005).

## Conflict of Interest Statement

The authors declare that the research was conducted in the absence of any commercial or financial relationships that could be construed as a potential conflict of interest.
